# Comments on: Knowledge and practices of postmenopausal women regarding vitamin D and osteoporosis

**DOI:** 10.61622/rbgo/2026rbgo108L24

**Published:** 2026-10-13

**Authors:** Claudia Jacqueline Rios Avalos, Billy Emanuel Rodriguez Cabrejo, Guillermo César Fernández

**Affiliations:** 1 Universidad Privada San Juan Bautista Facultad Ciencias de la Salud Escuela Profesional de Medicina Chorrillos Lima Perú Escuela Profesional de Medicina, Facultad Ciencias de la Salud, Universidad Privada San Juan Bautista, Chorrillos, Lima, Perú

Dear Editor,

We have read the article bi Silva el al. entitled "Knowledge and practices of postmenopausal women regarding vitamin D and osteoporosis", in which the authors evaluate the knowledge and practices related to vitamin D and osteoporosis in postmenopausal women.^(1)^ We believe that this study addresses an important public health issue. Below, we would like to make some comments on the work.

First, we observed a significant difference between the high use of vitamin D supplementation and the low level of knowledge among participants about this vitamin in relation to osteoporosis. Although most women reported taking supplements, many had difficulties identifying dietary sources, synthesis mechanisms, and benefits. This highlights a treatment model in which patients do not possess the necessary information to understand their health condition. This discrepancy between the frequent use of supplements and limited knowledge about vitamin D has also been described in other studies. O’Connor et al. found that despite the increasing use of supplements, there is insufficient knowledge among the population regarding functions, sources, and benefits, emphasizing the need for educational programs to promote health care related to appropriate vitamin levels.^(2)^

Likewise, health literacy favors adherence to therapeutic recommendations, as mentioned by Berkman et al., who point out that low literacy is associated with poorer adherence and worse health outcomes.^(3)^ On the other hand, the authors reported that of all participants, 55.38% had completed primary education or incomplete secondary education, and 16.92% had incomplete primary education or no education. This high percentage of participants with low educational levels could explain the limitations in understanding information about vitamin D supplementation and osteoporosis prevention. The WHO notes that health literacy is an important determinant for informed decision-making and the adoption of healthy behaviors.^(4)^

Another relevant aspect was the high use of photoprotection measures among participants. The authors reported that 68.46% of participants used sunscreen and 55.77% used physical barriers. This could contribute to reduced cutaneous synthesis of vitamin D, despite living in a region with high sun exposure. In relation to this, Passeron T et al. reported that brief and controlled sun exposure is necessary to ensure adequate vitamin D production, particularly in at-risk populations.^(5)^ However, the American Academy of Dermatology notes that there is no clearly safe level of ultraviolet radiation exposure to induce vitamin D synthesis without increasing the risk of skin cancer. For this reason, to maintain sufficient vitamin D levels, supplementation and diet are suggested over excessive sun exposure.^(6)^ In this context, we consider it important to provide individualized guidance to patients on safe sun exposure, vitamin D supplementation, and osteoporosis prevention through consultations with specialties such as gynecology, endocrinology, and dermatology. In this way, good bone and metabolic health would be promoted without increasing the risk of dermatological diseases.

In conclusion, the study by Bastos-Silva et al. provides valuable evidence on the degree of knowledge related to vitamin D and osteoporosis in postmenopausal women. Nevertheless, we consider that factors such as access to specialized services, health literacy, educational strategies, and the balance between photoprotection and sun exposure deserve broader discussion due to their possible impact on therapeutic adherence and the prevention of osteometabolic diseases.

## Data Availability

The research data are described in the letter presented.
